# Approaches to Preventing Intrapartum Fetal Injury

**DOI:** 10.3389/fped.2022.915344

**Published:** 2022-09-23

**Authors:** Barry S. Schifrin, Brian J. Koos, Wayne R. Cohen, Mohamed Soliman

**Affiliations:** ^1^Department of Obstetrics and Gynecology, Western University of Health Sciences, Pomona, CA, United States; ^2^Department of Obstetrics and Gynecology, David Geffen School of Medicine, University of California, Los Angeles, Los Angeles, CA, United States; ^3^Department of Microbiology, Immunology, and Molecular Genetics, University of California, Los Angeles, Los Angeles, CA, United States; ^4^Department of Obstetrics and Gynecology, University of Arizona College of Medicine, Tucson, AZ, United States

**Keywords:** cardiotocography, excessive uterine activity, fetal compensatory responses, fetal monitoring, fetal neurological injury, fetal hypoxia-ischemia

## Abstract

Electronic fetal monitoring (EFM) was introduced into obstetric practice in 1970 as a test to identify early deterioration of fetal acid-base balance in the expectation that prompt intervention (“rescue”) would reduce neonatal morbidity and mortality. Clinical trials using a variety of visual or computer-based classifications and algorithms for intervention have failed repeatedly to demonstrate improved immediate or long-term outcomes with this technique, which has, however, contributed to an increased rate of operative deliveries (deemed “unnecessary”). In this review, we discuss the limitations of current classifications of FHR patterns and management guidelines based on them. We argue that these clinical and computer-based formulations pay too much attention to the detection of systemic fetal acidosis/hypoxia and too little attention not only to the pathophysiology of FHR patterns but to the provenance of fetal neurological injury and to the relationship of intrapartum injury to the condition of the newborn. Although they do not reliably predict fetal acidosis, FHR patterns, properly interpreted in the context of the clinical circumstances, do reliably identify fetal neurological integrity (behavior) and are a biomarker of fetal neurological injury (separate from asphyxia). They provide insight into the mechanisms and trajectory (evolution) of any hypoxic or ischemic threat to the fetus and have particular promise in signaling preventive measures (1) to enhance the outcome, (2) to reduce the frequency of “abnormal” FHR patterns that require urgent intervention, and (3) to inform the decision to provide neuroprotection to the newborn.

“*To wrest from nature the secrets which have perplexed philosophers of all ages, to track to their sources the causes of disease, to correlate the vast stores of knowledge, that they may be quickly available for the prevention and cure of disease—these are our ambitions*”.

William Osler, 1889

“*How much do we know at any time? Much more, or so I believe, than we know we know!*”

Agatha Christie—The Moving Finger

## Introduction

Despite a half-century of widespread deployment, considerable debate remains about the clinical value of electronic cardiotocography (CTG) and the mechanisms by which abnormal fetal heart rate (FHR) patterns occur (1–3). Cited weaknesses of the technique include subjective interpretation of tracings, poor intra- and inter-observer agreement, insufficient sensitivity and specificity, and poor correlation with fetal acidemia (4, 5). Using current classifications of patterns, with their contradictions (6), CTG has not seemed to reduce the overall incidence of permanent intrapartum-linked neurological injury, although it may have lowered the incidence of neonatal hypoxic-ischemic encephalopathy (HIE) and seizures (7, 8). Long-term studies suggest a temporal improvement in outcome with the introduction of enhanced CTG techniques, but cannot attribute the improvement specifically to CTG (9).

We propose here to discuss the limitations of current classifications of FHR patterns and management guidelines based on them. We argue that current formulations of FHR patterns based on the detection of systemic fetal acidosis/hypoxia are counterproductive, both clinically and for the purpose of designing computer-based algorithms (10). Further, we believe that too little attention has been paid to the pathophysiology of FHR patterns, the provenance of fetal neurological injury, and the relationship of intrapartum injury to the condition of the newborn.

## The Premise of EFM

Since its inception, CTG has been used as a screening test for detecting fetal acidemia sufficient to warrant intervention, but insufficient to cause injury (11). Indeed, considerable experimental and clinical evidence has associated abnormal CTG patterns with low umbilical artery pH (UApH) and various adverse short- and long-term difficulties (12). There is a universal agreement on a relationship between acidemia and adverse outcome and between FHR patterns and acidemia and adverse outcome, but no evidence that information provided to the clinician or the computer scientist can be used to improve outcomes (1, 10).

In 2003, the American College of Obstetricians and Gynecologists (ACOG) required a very low scalp or umbilical arterial blood pH and depressed Apgar score before a causal relationship could be established between an intrapartum event and a subsequent brain injury (13). The subsequent ACOG publication of 2014, while still emphasizing the importance of pH at birth, affirmed that the evolution of CTG patterns could provide helpful insights (14).

Many fetuses neurologically injured during labor do not show severe metabolic acidosis at birth and may even be asymptomatic (15–17). On the other hand, most fetuses with even severe abnormalities of BD (<15) (18) do not require assistance at birth (2). The ACOG guidelines notwithstanding, an abnormal pH at birth, does not provide assurance of the intrapartum timing of the fetal neurological injury (Jonsson). This is a significant problem for those providing neonatal hypothermia for suspected HIE where the therapy must be instituted within 6 h of the presumed onset of the injury. Guidelines based on the detection of hypoxia or acidosis and the potential need for intervention have very limited sensitivity and specificity for identifying the fetus at risk or enhancing the outcome. Acid-base parameters, therefore, should not be used as an exclusive surrogate to assess the mechanism and timing of intrapartum injury or optimally direct therapy. Nor should they be the basis for developing computer-based algorithms for clinical management (3, 16, 19–23).

In a retrospective cohort study of 29,787 terms, singleton, non-anomalous births between 2012 and 2020, Johnson and colleagues found a weak to absent correlation between UApH and BD and both 1- and 5-min pgar scores for all pH ranges. The authors then argue that these data undermine one of the rationales for using the CTG, concluding that “No amount of modification of CTG interpretation to better predict UApH is likely to lead to improved newborn outcomes.”

As pointed out by Levene and Chervenak, obtaining a pH tells you about things that can be measured easily (the pH and BD), but nothing about fetal blood pressure (BP) or the adequacy of cerebral blood flow (CBF) (24). We will offer evidence that the CTG, properly interpreted, permits inferences about fetal BP and CBF.

## The Precepts of Monitoring

Irrespective of their relationship to fetal systemic acidemia, current formulations of FHR features, as a group, do not consider non-hypoxic sources of brain injury, such as infection, trauma, or ischemia from head compression or stroke or injury preceding labor. They also fail to consider CTG-provided insights into fetal behavior or excessive uterine activity (EUA) (25, 26), and do not use the fetus as its own control or evaluate the evolution of fetal adaptive responses to various stressors present during labor (27–29).

To prevent adverse outcomes from the events of labor, the harm (injury) must be preventable. Implicit in the Harir study and numerous other studies is the notion that the normal CTG tracing at the outset of labor represents both neurological integrity and the absence of any hypoxic or ischemic threat. We will attempt to define the “normal” tracing and the evidence for this precept but first, offer a clarification of certain terms.

The *baseline* FHR refers to the average, stable FHR excluding decelerations and accelerations over a period of time during labor. The *basal* FHR is the baseline rate established at the onset of labor during a period of fetal quiescence. The relationship of the baseline rate to the basal rate assists in understanding trends in the FHR; it uses the fetus as its own control eliminating the notion of a “baseline” rate as the “current baseline rate” irrespective of the provocations acting upon it. Thus, tachycardia and bradycardia, customarily identified by an absolute value (i.e., >160 bpm or <110 bpm), should also be diagnosed according to changes relative to the basal rate (i.e., +/– 15 bpm) (30).

Sympathetic and parasympathetic inputs from the autonomic nervous system to the sinoatrial node regulate the baseline FHR and its variability. These can be modified by a number of physiological and pathological influences including gestational age, medications, fetal activity, and maternal core temperature (31). Normal baseline variability results from the unpredictable changes in the R-R interval from beat-to-beat (“short-term variability”) superimposed on the broader peak-to-trough oscillations of the baseline measured over perhaps a minute or more (“long-term variability”). Normal baseline variability and a stable rate in the normal range (not interpreted during accelerations or decelerations) denote appropriate autonomic control over the FHR and cardiac output. An ability to rapidly adjust cardiac intervals (primarily due to efferent parasympathetic activity) (32) is a normal feature of cardiovascular homeostasis. Although moderate variability is widely believed to be the most important feature of normal oxygenation, consideration of the stability of the baseline, and the presence of decelerations will permit a more comprehensive approach to the physiological state of the fetus (33).

During labor, the baseline FHR can only be established when the rate is stable between each contraction or following each deceleration, especially during the second stage. The inability to affirmatively detect a stable baseline rate over several consecutive contractions represents an “undefined” or “indeterminate” baseline and is a risk factor for adverse outcomes (34).

## Normal CTG Pattern—Variability, Accelerations, and Behavioral Cycles

**Figure 1:**
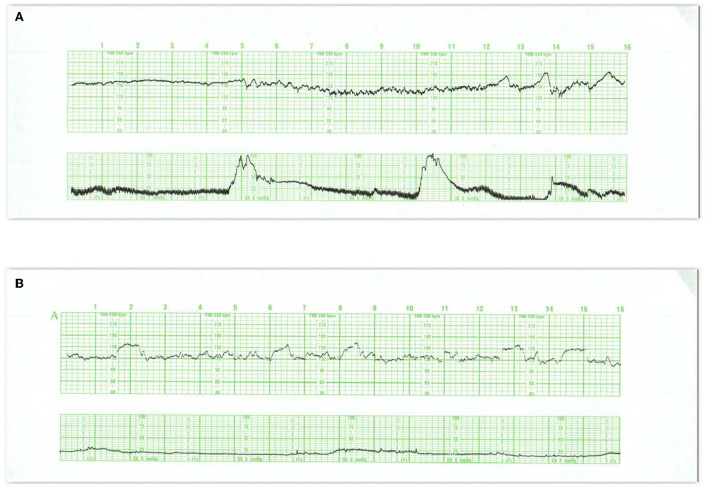
These tracings illustrate state changes in the fetus during early labor. Notice the three distinct FHR patterns in **(A)**. In the first 5 min the pattern is characterized by diminished short- and long-term variability representing quiet sleep. From 5M to 12M, the short- and long-terms variability increases and the rate decreases, likely representing fetal breathing movements, with accelerations. From 12M to 16M the tracing becomes reactive with normal variability, obvious accelerations, and presumable, fetal movement. This is a reassuring tracing and would be no more troublesome if the baseline rate were below 120 or 110 bpm (a baseline bradycardia). **(B)** Obtained with a direct scalp electrode reveals accelerations during and between contractions. These accelerations are likely associated with fetal movement. Fetal reactivity may persist into early or mid-labor despite the administration of analgesia. Scaling−30 bpm/cm—vertical, 3 cm/min—horizontal—each panel is 16 min wide.

**Figure 2:**
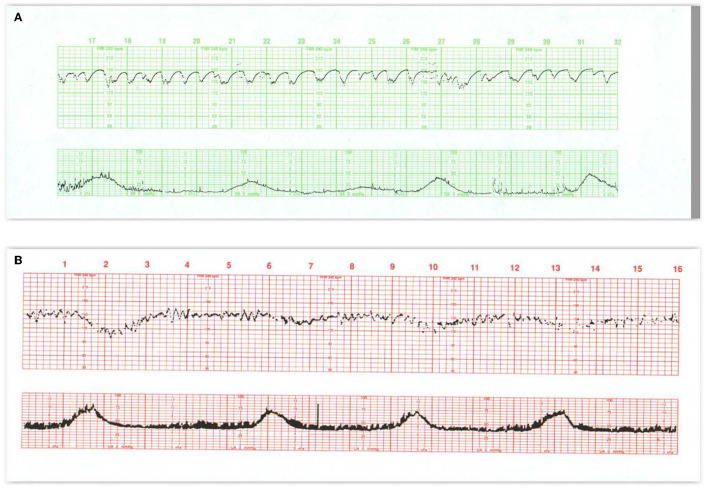
**(A)** Represents an exaggerated “scallop” pattern, probably associated with fetal sucking and decreased fetal movement. This is a benign behavioral pattern. **(B)** Tracing invites classification as a positive admission test. The panel contains unequivocal decelerations that appear late in timing, but there is significant variation in the onset and duration of the decelerations. The baseline variability is moderate and shows no tendency to tachycardia or decreased variability. The UC channel reveals high frequency, low amplitude, somewhat irregular spikes which represent fetal breathing movements. When fetal breathing movements cease, the decelerations disappear. A benign tracing. Scaling−30 bpm/cm—vertical, 3 cm/min—horizontal—each panel is 16 min wide.

In the normal term and near-term fetus, periods of lesser, even absent, variability, and greater variability recur in cycles lasting up to 40 min (Figures 1A,B) Accelerations are usually associated with individual fetal movements, which are occasionally sustained (Figure 2A) and connote normal neurological behavior. Fetal hypoxemia or acidosis has never been reported in the presence of this cyclic pattern (35). These criteria for cyclicity are one of the cornerstones of the assessment of normal behavior in both the fetus and newborn. Nijhuis and ten Hof (36) Normal behavioral responses to contractions and the associated increased fetal BP include increased variability, accelerations with fetal movements at the outset or during contractions, or even brief decelerations (37–39). Decelerations mimicking late decelerations have also been reported in association with the emergence of fetal breathing movements toward the end of the contraction (Figure 2B) (37, 40) With either response, baseline rate and variability are maintained. Accelerations tend to disappear as labor advances and the fetal head descends, sometimes with the appearance of decelerations. This represents an increasing dominance of the parasympathetic input associated with head compression. Accelerations persist if the fetus is in breech presentation or following the administration of atropine (a parasympathetic blocker) to the mother (41).

The frequency of accelerations immediately prior to the delivery of a severely hypoxic fetus in the Hehir paper cited above does not comport with other literature and invites the inference that it was the maternal heart rate (MHR) pattern that was being recorded (see below) (42).

## Abnormalities of Baseline Features

**Figure 3:**
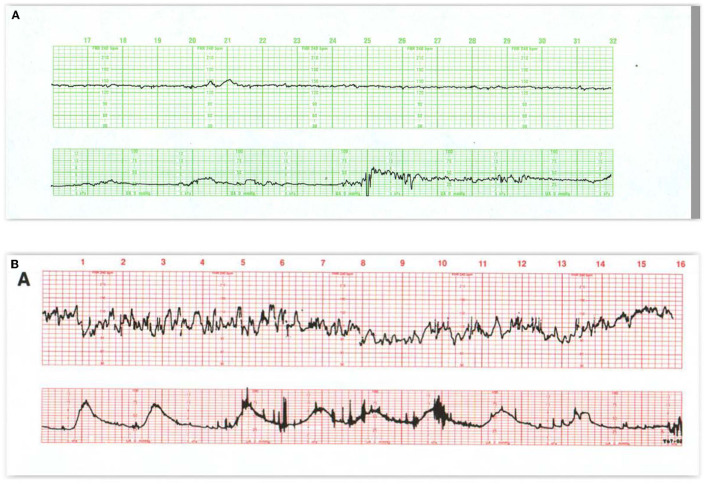
**(A)** This tracing, obtained in early labor, reflects several effects of medication on the FHR pattern and uterine contractions. Narcotics decrease baseline variability as well as the frequency and angularity of the accelerations associated with fetal movement. Notice that the accelerations appear only with contractions, if at all, and that the accelerations arise from diminished variability. In **(B)** contractions are frequent, variability is exaggerated (saltatory), and decelerations are absent. The fetus responds to the excessive uterine activity between 5M and 10M with an erratic but prolonged deceleration. As the contractions space out, the fetal heart rate begins to return and then exceed the previous baseline for a brief period which is proportional to the duration of the fetal hypoxemia. Often the fetus exhibits one or more late decelerations during the recovery (at 12M). The nadir of the deceleration never goes below 90 bpm and the fetus retains considerable variability during the deceleration. Clinical management should include cessation of oxytocin, repositioning to the left side, and, if necessary, administration of tocolytics to effectively diminish the uterine activity and promote optimum maternal-fetal circulatory exchange. Scaling−30 bpm/cm—vertical, 3 cm/min—horizontal—each panel is 16 min wide.

Reduced or absent FHR variability can signal acidosis (43, 44); however, when associated with cyclic episodes of moderate variability and accelerations, they commonly reflect normal fetal sleep. Diminished variability (without decelerations) is also a common, self-limited response to ataractic medication. In this circumstance, diminution in variability appears before accelerations disappear (Figure 3A). Sustained, completely absent variability without decelerations is uncommon, but generally suggests brain pathology, severe drug effect, fetal growth restriction, anomaly, or arrhythmia (45). Persistently reduced or absent heart rate variability has been correlated not only with fetal hypoxia and asphyxia and neonatal HIE but also when associated with decelerations (*see* below). Reduced variability analyzed long after delivery has also been found in patients with autism spectrum disorder (ASD), cerebral palsy (CP), and epilepsy, but a direct connection to the intrapartum tracings has not been made (46).

Differentiating between reduced and absent variability can be misleading, and there would seem to be little practical value in making the distinction (43). It is the persistent trend away from moderate variability (and cyclicity) toward decreased or absent variability that is the relevant finding. The use of external devices may artifactually increase or decrease apparent variability, but they will not misrepresent the overall rate pattern (*see* below).

### Increased Variability

Increased FHR variability (Figure 3B) is not always more benign than reduced variability. It may be observed during the operative vaginal delivery or following the administration of ephedrine in support of maternal BP (47–52). It has been referred to as “saltatory” when sustained or “zig-zag” when brief, Tarvonen et al. (53) or simply, increased variability but is most often found in association with excessive uterine activity and variable or prolonged decelerations during expulsive efforts of the second stage. In the first stage it may anticipate late decelerations. It is an early, adverse sign that reflects an autonomic stress response to umbilical cord or head compression or to a mild reduction in oxygenation that develops before significant acidemia or hypotension (51). Increased variability can impede the determination of the true baseline FHR and should be considered as an indication to diminish the frequency and intensity of contractions and pushing efforts until it is resolved.

### Tachycardia

**Figure 4:**
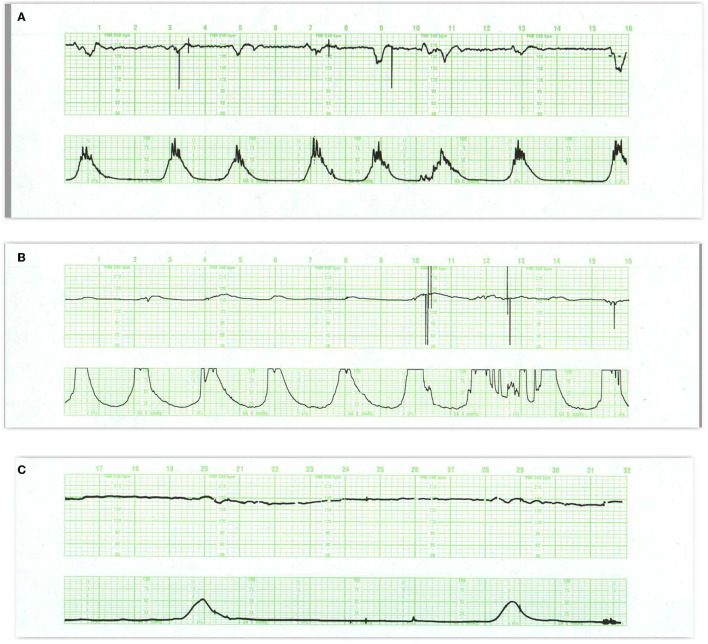
**(A)** Demonstrates baseline tachycardia and decreased absent short-term variability in an otherwise resilient fetus in a febrile mother. Under normal circumstances, when the baseline heart rate rises, both long- and short-term variability diminish. Notice the accelerations “overshoot” that tend to follow the trivial variable decelerations. Fetal monitor patterns may not be reliable signs of early fetal infection and approaches to treatment of fetal tachycardia vary. Despite the maternal fever, the tracing suggests a resilient infant. The newborn should be evaluated for sepsis. **(B)** Offers considerable insight into the signs of abnormal neurologic control of the heart rate pattern, not necessarily related to injury. The entire pattern takes place in the second stage of labor; contractions are frequent, and the mother is pushing with each. Despite this assault, the fetus maintains a perfectly stable baseline rate with some accelerations and no decelerations—features that preclude fetal asphyxia. **(C)** Reveals a markedly abnormal tracing including baseline tachycardia, small variable decelerations with overshoot, and some instability of the heart rate as reflected by the prolonged return to the baseline after the occasional contraction. It is implausible that the tracing immediately preceding this was normal. Scaling−30 bpm/cm—vertical, 3 cm/min—horizontal—each panel is 16 min wide.

Absolute or relative elevations of the baseline FHR during labor in the absence of decelerations are rarely due to fetal hypoxia. Common causes of tachycardia without decelerations include fetal manipulation (with exaggerated variability– usually lasting < 10 min), maternal fever, fetal infection, cardioactive drugs, and arrhythmias (Figure 4A). Tachycardia and absent variability may also reflect preexisting neurological injury (54) (Figures 4B,C). While the normal range of baseline FHR is considered to be 110–160 bpm, a basal heart rate above 150 bpm at term exceeds statistical limits and should be considered with suspicion unless accompanied by reassuring accelerations and moderate variability (52, 55, 56).

### Other Adverse Baseline Features

**Figure 5:**
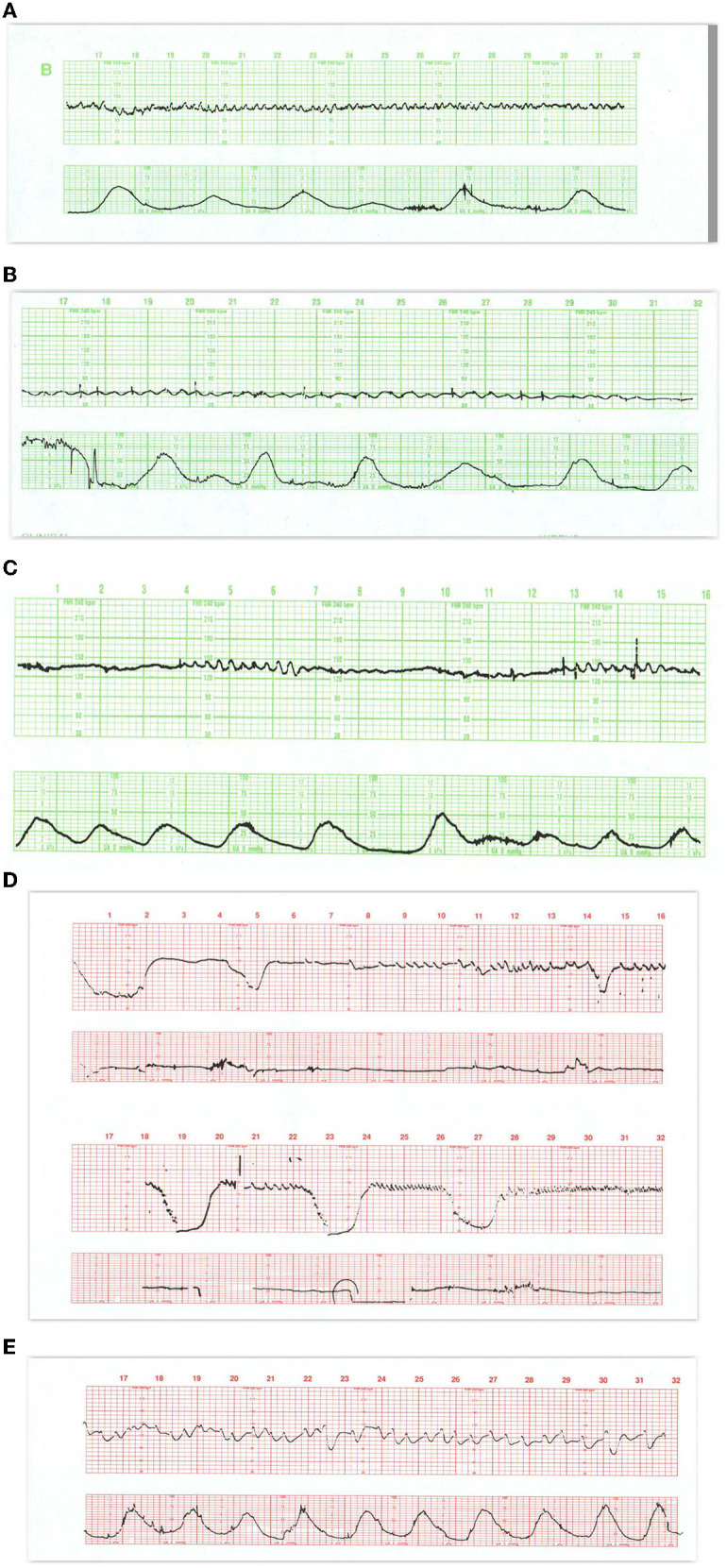
In **(A)** the appearance of regular oscillations in the baseline invites the designation “sinusoidal pattern.” In this instance, the somewhat, irregular oscillations tend to be peaked and seem to contain short-term variability. Such patterns may be seen with the normal rest-activity cycles in the healthy fetus and referred to as pseudosinusoidal. Indeed, other areas of the tracing reveal normal reactivity. In the presence of variability or reactivity, sinusoidal features may be dismissed. True sinusoidal patterns, even those developing after the administration of narcotics, tend to be smoother and less angular, and are associated with decreased baseline variability. **(B)** Reveals bradycardia with obvious oscillations in the pattern (“sinusoidal” in a severely anemic fetus. **(C)** Reveals absent fetal heart rate variability and episodes of sinusoidal pattern at 4M, 13M, and 18M that augur fetal death. The frequent uterine contractions from excessive oxytocin have no apparent impact on the compromised fetus. Notice the obvious arrhythmia starting at 23M. This unexpected, 1.5-min episode of bigeminal heart rate pattern heralds the onset of the bradycardia and fetal death. **(D)** Illustrates the evolution of the “saw-tooth pattern” in a neurologically injured fetus. The tracing begins most ominously with several prolonged decelerations associated with absent baseline variability. Beginning just after 6M, the baseline is punctuated by abrupt, brief upswings (about 10 bpm) followed by a more leisurely downslope. The duration of the entire deflection is < 10 s. From that point on, the frequency of these excursions increases dramatically and predictably. Between 9M and 10M they appear with a frequency of 3–4/min. By 16M they appear with a frequency of 4–5/min. By 28M the frequency is simply too high to count. In addition, the amplitude decreases somewhat as the frequency increases. The overall progression of this baseline pattern should reveal how different this pattern is from normal baseline variability. Normal variability is random and unpredictable. **(E)** Reveals a “checkmark” pattern representing deterioration in a fetus with a preexisting cerebral injury. It is difficult to define any consistent pattern of accelerations or decelerations and should not be confused with normal long-term variability. In addition, the baseline rate is falling in the face of excessive uterine activity. These features anticipate the ultimate death of the newborn. Scaling−30 bpm/cm—vertical, 3 cm/min—horizontal—each panel is 16 min wide.

The sinusoidal FHR pattern, originally described in anemic fetuses (57) and more recently in association with HI injury and sepsis (58), is a variant of absent beat-to-beat variability upon which is superimposed regular, predictable oscillations with a frequency of 2–5 cycles/min and an amplitude of 5–15 bpm. It occurs in the absence of a normal FHR pattern nearby. Various formulations of sinusoidal patterns have been promulgated (59–61) (Figures 5A–C). Ominous sinusoidal patterns are generally prolonged with fetal anemia, severe hypoxia, or sepsis. They sometimes occur transiently in association with adverse FHR recovery from variable decelerations.

Pharmacologic manipulations can elicit FHR patterns that do not ordinarily appear in the intact fetus and may briefly mimic signs of neurological injury or anomaly. For example, maternal narcotic administration can reduce variability and elicit a harmless pattern mimicking the sinusoidal pattern, which is termed “pseudosinusoidal” (Figure 5A). In experimental animals, the administration of arginine vasopressin (AVP) induces a transient sinusoidal pattern (60).

Other persistent baseline features that carry ominous import are the sawtooth and checkmark patterns (Table 1). Once recognized, they demand immediate evaluation and possible intervention because of their association with asphyxia and subsequent harm (Figures 5D,E) (62–65). It is important to emphasize that these patterns (in the absence of a normal pattern nearby) are not markers of asphyxia, but markers of brain injury.

**Table 1:** Electronic fetal monitoring definitions.

Basal heart rate	• The baseline fetal heart rate fixed at the beginning of labor with fetus quiescent.
**Baseline Heart Rate**	The stable fetal heart rate between contractions at any time during labor. The average stable heart rate measured between contractions generally over 10 min.
Deceleration recovery	• The response of the FHR to a deceleration
Normal recovery	• Return to the previously normal baseline rate and variability
**Adverse Features**	
Sinusoidal pattern	• Visually apparent, smooth, sine wave-like undulating pattern in FHR baseline with a cycle frequency of 3–5 per minute. Occurs in the absence of normal FHR pattern nearby. May be brief or persistent.
Checkmark pattern	• Unique pattern seen in decompensated fetus revealing a jagged (zig-zag) pattern.
Sawtooth pattern	• Rapid, high frequency (20+cpm), low amplitude (< 15 bpm), peaked oscillations in the heart rate that generally increase in frequency and decrease in amplitude over time. Associated with fetal neurological injury.
Conversion pattern	• A CTG pattern in which there is a dramatic change in rate, variability and pattern of deceleration within 1–2 contractions—suggests fetal ischemic injury.
Overshoot	• An acceleration of the FHR immediately following a variable deceleration with duration proportional to the amplitude of the preceding deceleration. Usually associated with alterations in baseline rate and variability.
Delayed return	• A slow return to the baseline from the nadir of a variable deceleration.
Peaked return	• An abrupt peak at the end of a variable deceleration followed by a late deceleration. An ominous commentary usually leading to fetal death.

bpm, beats per minute; cpm, cycles per minute; CTG, cardiotocographic; FHR, fetal heart rate.

**Figure 6:**
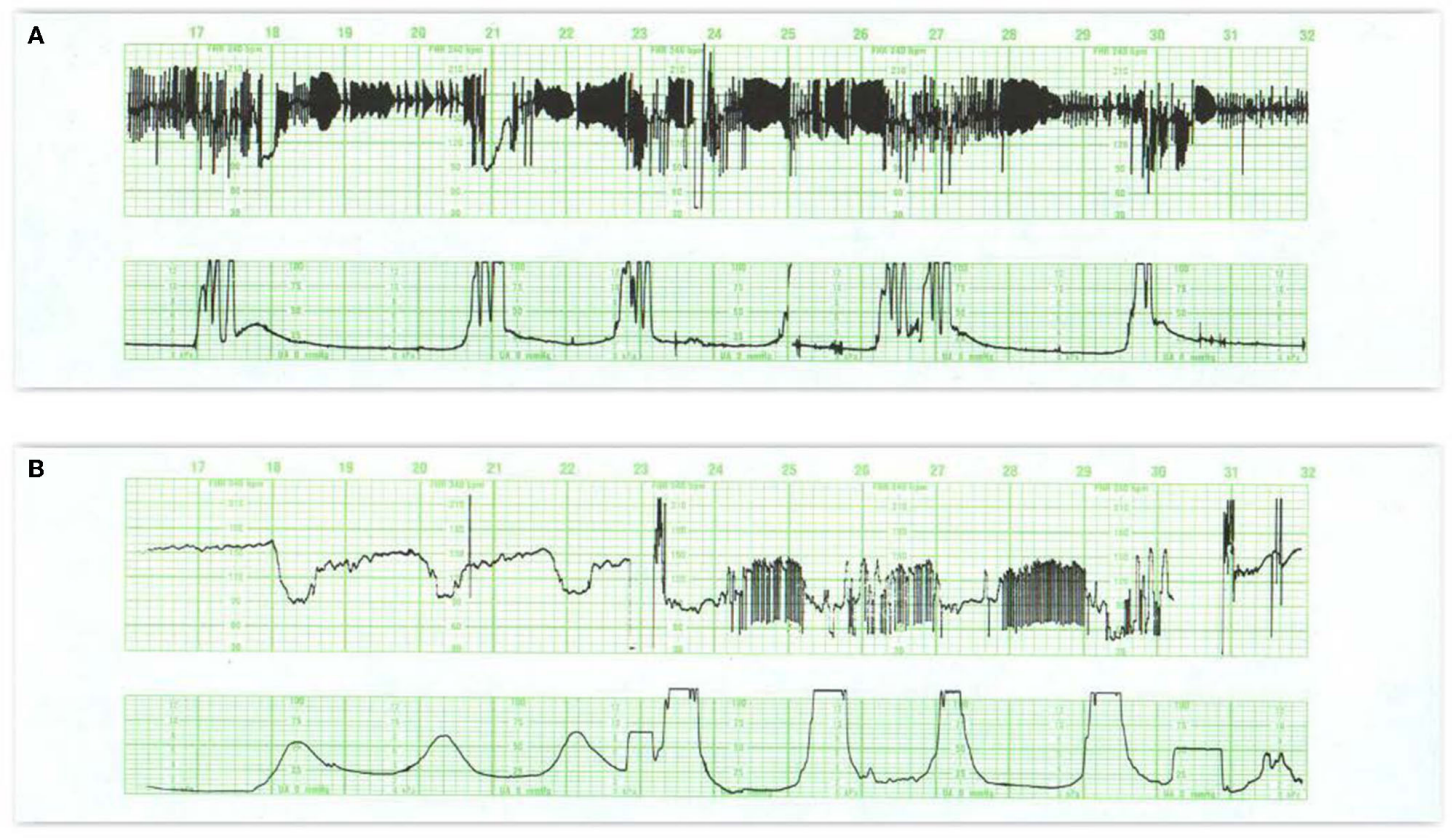
**(A)** Reveals a fetal arrhythmia—not artifact. Note the recurrent, symmetrical geometric patterns produced by clusters of ectopic beats. This pattern results from cardiac bigeminy (alternating intervals), with the shorter interval getting progressively longer and the longer interval getting progressively shorter. Notice that all of the changes are contained within a narrow range of heart rates (really intervals). These ectopic ventricular beats are multifocal (different heights, different patterns) and frequently alternate with normal beats. Notice the occasional decelerations at 18M and 21M. Although the tracing reveals a stable heart rate and probably inconsequential variable decelerations, the frequency of the ectopic beats precludes estimation of the amount of beat-to-beat variability. This tracing mandates ultrasound examination of the fetus to rule out hydrops or congenital anomaly. A pediatrician should be present in the delivery room to assist with any resuscitation. **(B)** Illustrates variations on the theme of second stage decelerations. Variable decelerations appear with each contraction. After the onset of pushing (24M), the decelerations are followed by an arrhythmia at the end of the deceleration. These excursions are restrained, limited, create geometric (bigeminal) patterns, and are restricted to the period between contractions. In this situation, the patient should refrain from pushing until the character of the heart rate pattern has defined itself clearly.

Some CTG patterns represent fetal arrhythmias (66), which can be best detected by a direct scalp electrode or echocardiogram. The most common are atrial premature beats and are almost invariably benign (Figures 6A,B). Ventricular ectopy is also seen frequently and probably reflects the immaturity of the conduction system rather than a response to hypoxemia. Occasional cases of supraventricular tachycardia and of bradycardias related to various degrees of heart block have been described and can be associated with cardiac failure.

It is important to emphasize that during labor changes in baseline rate and variability unrelated to decelerations are unlikely to represent fetal hypoxia, but rather are physiologic (sleep, response to medication, etc.) or pathological, i.e., secondary responses related to the duration and severity of the hypoxic/ischemic challenge.

## Decelerations

FHR decelerations are common during labor (67) and are a response to systemic or regional oxygen deprivation caused by impaired umbilical, uteroplacental, or cerebral blood flow (68). In the presence of hypoxemia, decelerations emerge in the fetus long before there is any change in the baseline rate, variability, or pattern of recovery from decelerations (69) and before acidemia is detectable. Table 2 describes the characteristics of the various deceleration patterns. Notice the distinctive provenance and pattern of decelerations related to the short-term impairment of umbilical or cerebral blood flow (intrinsic) vs. those associated with impairment of utero-placental blood flow (extrinsic). This distinction assists in determining the provenance of decelerations (e.g., head compression) and the potential benefits of therapeutic interventions.

**Table 2:** Characteristics of decelerations.

**Intrinsic—related to impaired fetal blood flow** **Early deceleration**—Caused by increased intracranial pressure Symmetrical, gradual onset from beginning of contraction, repetitive, contained within contraction, proportional to contraction Not associated with hypoxia Evolves into variable decelerations in the second stage of labor. Seen primarily late first stage of labor, 2nd stage of labor, malposition Severity measured by response of baseline heart rate (tachycardia / variability) or prolongation of deceleration beyond contraction.	**Variable deceleration**—Caused by cord or head compression Variable onset in relation to contraction, usually abrupt fall and abrupt return—may not repeat May have brief accelerations before or after deceleration (“Shoulder”) Threshold effect—not associated with hypoxia with normal recovery Severity measured by: Duration, amplitude and impact on baseline rate and variability Recovery from deceleration—prolonged, overshoot, exaggerated variability	**Extrinsic—related to impaired uterine blood flow** **Late deceleration** Onset— after onset of contraction, recovers beyond end of contraction Symmetrical, proportional in amplitude and duration to contractions, repetitive Threshold effect—Associated with hypoxemia Severity measured by impact on baseline rate and variability	**Extrinsic / Intrinsic—severe** Prolonged deceleration Onset—any time—frequently after variable deceleration, excessive uterine activity, spinal anesthesia, maternal body cooling. Usually abrupt fall in rate—slower drop with chronic hypoxia—agonal event Threshold effect—the longer the duration the greater the risk of acidosis / adverse outcome Severity measured by preceding FHR pattern, pattern during deceleration, duration and recovery With severe hypoxic / ischemic event—does not permit diagnosis of injury until after recovery

### Deceleration Recovery

While the vast majority of decelerations are associated with normal outcomes, decelerations accompanied by changes in the baseline rate (tachycardia or bradycardia) or variability (decreased or increased) are associated with a higher risk of various adverse outcomes (52). Those patterns that retain normal baseline features between decelerations generally have good outcomes.

As a clinical strategy, therefore, the deceleration waveform (including timing, symmetry, depth, and duration) should be used to identify the likely provenance of the impaired blood flow. The recovery and the exuberance of the post-deceleration responses provide early insight into the adaptive responses and reserves of the fetus attempting to return to cardiovascular homeostasis. Adverse heart rate responses to late decelerations include a rise in baseline rate and a decrease in variability. The recovery from a variable deceleration, on the other hand, may display one of the several patterns according to the severity, duration, and frequency of the decelerations (Table 3).

**Table 3:** Uterine activity parameters.

**Contraction parameter**	**Average**	**Excessive**
Frequency	2–4.5 UC / 10 min.	>5/10 min (x2)
Intensity	25–75 mm Hg	Not defined!
Duration	60–90 s	>90 s
Resting Tone	12–20 mm Hg	>20 mm Hg
Interval between peaks	2–4 min.	<120 s
Rest time^*^	50–75%	<50%
Montevideo Units -	Not clinically useful	

Rest time—interval when contractions and pushing are absent.

BPM, beats per minute; FHR, fetal heart rate; mm Hg, millimeters of mercury. Modified in part from (44).

The importance of adverse features following variable decelerations has been de-emphasized because no differences occurred in their frequency between acidemic (UA pH <7.10) and non-acidemic newborns (28). Nevertheless, they are important early responses and should prompt efforts at deploying conservative interventions before the development of fetal compromise and before the exhaustion of critical cardiovascular adaptations and the deleterious effects of systemic acidemia.

Appropriate responses to impaired deceleration recovery usually involve lateral positioning, decreasing pushing efforts, but especially reducing the frequency and intensity of uterine contractions to allow recovery between them (70). In addition, augmenting maternal inspired oxygen concentration and correcting maternal hypotension are valuable in some circumstances. These measures can restore fetal reserves and obviate the need for intervention.

### Early Decelerations

**Figure 7:**
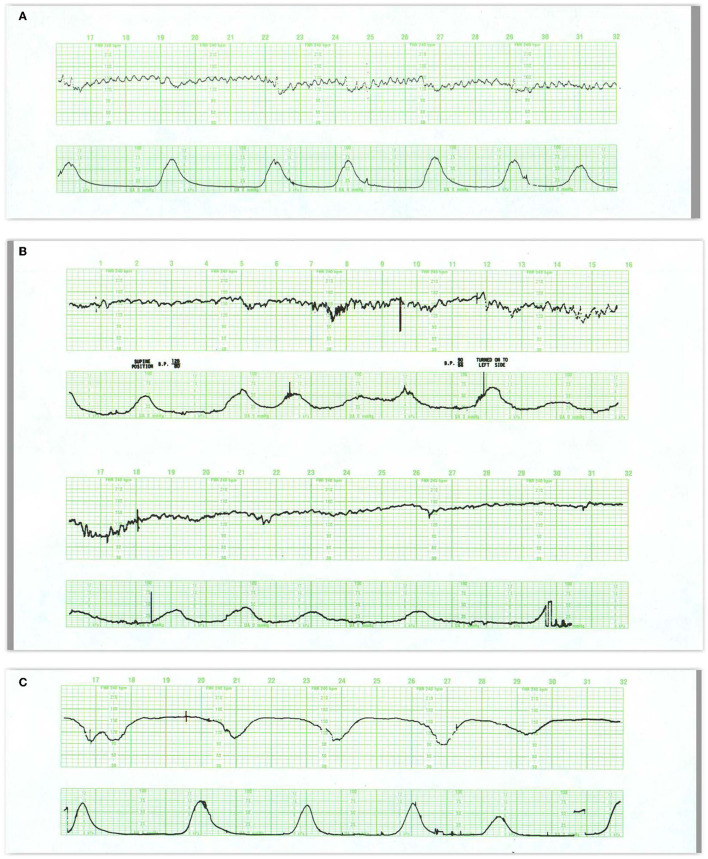
**(A)** This tracing reveals regular, predictable oscillations interrupted by mild (early) decelerations. These patterns are quite benign and require no intervention. They do represent fetal head compression with variable decelerations when pushing starts in the second stage. **(B)** Illustrates the development of late decelerations following epidural anesthesia and maternal hypotension. Notice the increased frequency of contractions immediately after the epidural. During recovery, there is a rise in the baseline and a diminution in the baseline variability along with some decrease in the frequency of contractions. The decelerations should be managed by the administration of oxygen, lateral positioning, hydration, and diminution of oxytocin infusion. At the same time, it is reasonable to presume that in this previously normal fetus, these maneuvers will indeed correct the transient episode of distress. Pre-hydration and the patient's maintenance of a lateral position after the anesthesia will dramatically reduce the incidence of late decelerations. **(C)** reveals late decelerations and absent variability. This tracing is quite ominous, revealing a combination of acute distress, late decelerations, and chronic distress with a flat heart rate baseline and slight tachycardia. Immediate preparations for delivery are required. While conservative maneuvers such as oxygen, lateral position, and hydration are appropriate, they are unlikely to ameliorate the fetal condition. Oxygen may reduce or eliminate the late decelerations but not improve the fetal condition. Before deciding to continue with the labor, oxygen should be discontinued. If decelerations reappear and contraindications are absent, the fetus should be delivered expeditiously. If decelerations do not reappear after oxygen removal, then the acute distress has been alleviated and an intelligent decision must be made about the chronic distress pattern. Given the stable baseline rate and absent variability, we may reasonably infer that the monitoring was begun long after the onset of asphyxia. Scaling−30 bpm/cm—vertical, 3 cm/min—horizontal—each panel is 16 min wide.

Early decelerations (Figure 7A) are included in some classifications of normal FHR patterns, likely because they are not associated with systemic oxygen deficiency. Nevertheless, they signal a potential threat to the fetus and should not be considered totally benign. Early decelerations reflect increased ICP due to head compression which transiently and recoverably reduces brain blood flow and oxygen delivery when compensatory responses are adequate. They usually occur in the first or early second stage of labor—after membrane rupture and prior to the onset of pushing. With the descent of the fetal head and the maternal expulsive efforts, even greater pressure is exerted on the fetal head resulting in decelerations that may extend beyond the contraction or may transition in appearance to variable decelerations (71, 72).

### Late Decelerations

The morphology of a late deceleration (Figures 7B,C) roughly mirrors that of the contraction (and the associated fall in placental oxygen transfer) but with a delayed onset. The lag time of the deceleration after contraction onset reflects the fetal oxygen reserves and circulation time, as well as the time for the fall in arterial oxygen tension required to activate the aortic O_2_ sensors. Late decelerations are always proportional in size to the duration and amplitude of the underlying contractions. Accelerations are never part of the late deceleration waveform which remains consistent irrespective of any rise in baseline or loss of variability or neurological injury. There is no required minimal amplitude for late decelerations, although, when shallow, they are more readily detected when the baseline variability is diminished.

If the limitation of oxygen availability to the fetus brought about by a uterine contraction is self-limited, the rise in fetal BP and sympathetic output accompanying the contraction permit the baseline rate and variability as well as the acid-base balance to remain stable. Further contraction-induced falls in uteroplacental blood flow and oxygen delivery will evoke additional compensatory responses following the decelerations that include a rise in baseline rate and a decrease in variability as lactic acidemia develops. Self-limited causes (e.g., epidural- associated hypotension) and the initiation of recovery measures (e.g., maternal position change) will generally lead to the disappearance of decelerations and then a return to a normal baseline rate and variability without incurring significant compromise or neurological injury. With continued progression, the pH falls further, and the baseline rate eventually attains a fixed tachycardia (typically 160–180 bpm) with absent variability. Continuing hypoxia with severe acidemia eventually exhausts fetal compensatory reserves, eliciting a cascade of hypotension, unstable FHR baseline, less distinct (or disappearance of) decelerations, bradycardia, and fetal demise. Along this advanced trajectory, the fetus may sustain neurological injury, typically cortical white matter injury, and an acute injury pattern on neuroradiological examination. This sequence is predictable

from the outset and is amenable to timely intervention to improve uteroplacental perfusion or to affect delivery.

### Variable Decelerations

**Figure 8:**
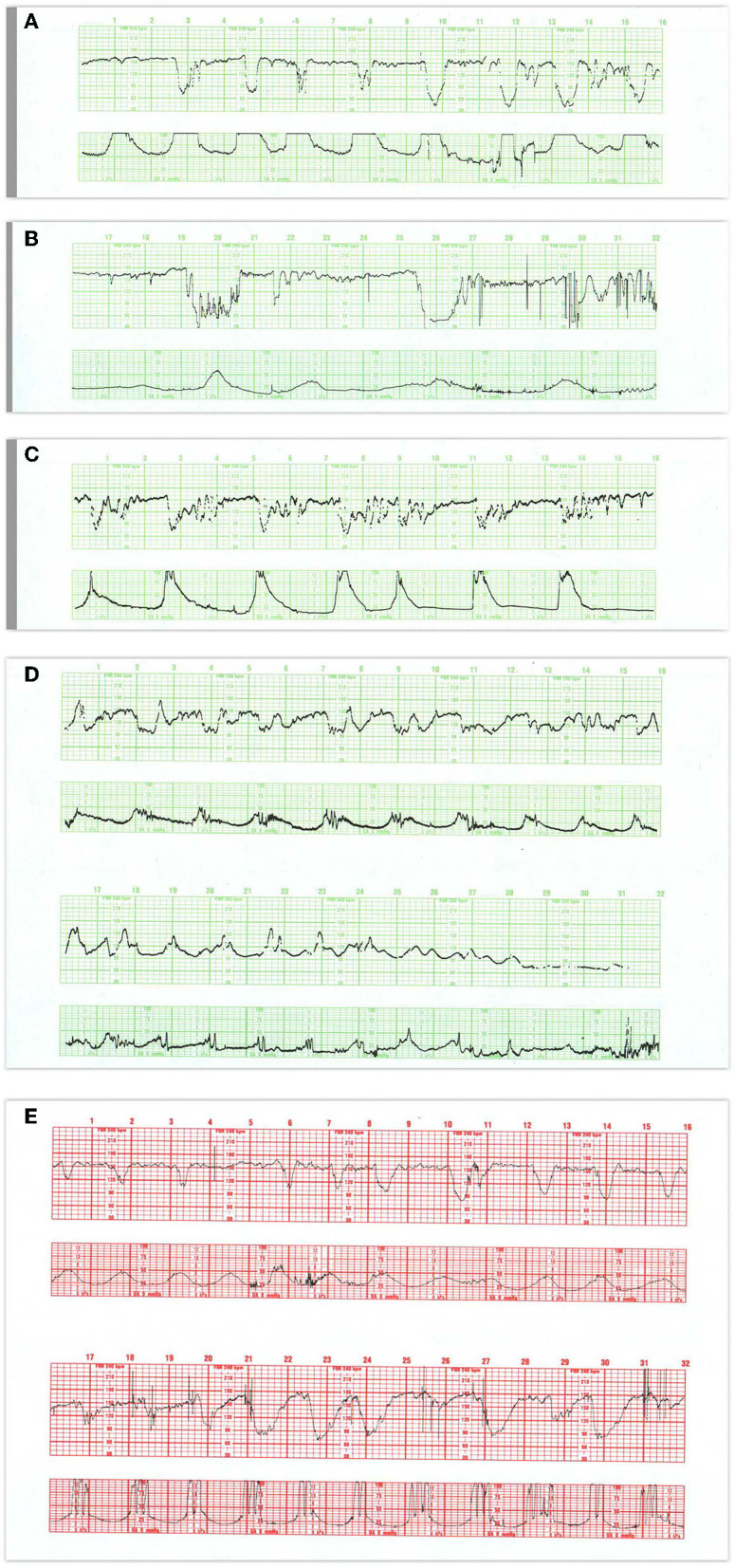
**(A)** Demonstrates the typical FHR pattern associated with occiput-posterior positions during the second stage of labor. The tracing is dominated by recurrent, variable decelerations, some of which reach 60 bpm and develop transient, nodal rhythm (9M, 11M, and 13M). Despite the frequent decelerations and contractions (10 per 16 min), the baseline rate remains quite stable without loss of variability. The frequent accelerations (“shoulders”) both initiate and follow the decelerations. Fetuses with occiput posterior positions tend to have far more frequent variable decelerations than those with occiput anterior position. The variable decelerations tend to be isolated rather than coalesced and usually return promptly to baseline on cessation of expulsive efforts. No intervention is required here but it is important to reduce the frequency of contractions and restrict expulsive efforts to those times at which the pattern has returned to normal after a previous deceleration. **(B)** Illustrates several features of variable decelerations including their intermittent nature, variable pattern, and co-association with variable accelerations (“shoulders”). Exaggerated long-term variability (saltatory) pattern is especially likely at the end of the deceleration. Many variable decelerations are anticipated by an acceleration followed by a rather abrupt downslope, occasionally culminating at 20M and 26M in a brief episode of asystole (heart block). Note the dramatic differences between the patterns at the bases of these two variable decelerations. In the deceleration at 20M, the heart rate stabilizes temporarily above 60 bpm and reveals exaggerated variability (probably some ectopic beats as well). In the deceleration at 26M, the heart rate stabilizes below 60 bpm and reveals the characteristic pattern of nodal rhythm, with its brief deceleration and subtle warmup. In both instances, the return to baseline is erratic but prompt, with rapid recovery of the baseline rate and variability. Thus, the pattern at the base of the deceleration carries little prognostic value but simply reflects the mechanism of cardiac pacing (nodal or sinus). These changes, in turn, reflect the level to which the rate descends. Thus, the variable deceleration at 26M is not “more severe” or “more ominous” than the deceleration at 20M. Occasionally, as in this patient after 27M, such exaggerated decelerations produce a small drop in the baseline rate but no real diminution in variability. These frequent decelerations early in labor suggest compression of the umbilical cord from occult prolapse, nuchal cord, or membranous insertion of the cord. While the tracing, strictly speaking, does not bespeak fetal deterioration or compromise, the likelihood of these decelerations recurring later on is considerable. This fetus was delivered vaginally; the placenta revealed membranous insertion of the cord. **(C)** Illustrates frequent, variable decelerations with “slow return to the baseline.” Despite the very frequent contractions and decelerations, the baseline heart rate remains stable with satisfactory and sometimes increased variability (saltatory pattern). These dramatic changes are quite frequent during the second stage especially in unmedicated labors. They frequently, but not always, moderate when pushing ceases. In this case, cessation of pushing, if anything, exaggerates the saltatory pattern. Saltatory patterns suggest compensation for previous episodes of stress, but probably not asphyxial stress. Intervention is unnecessary in this case and deterioration is uncommon with proper preventive care. **(D)** Illustrates recurrent, variable decelerations that deteriorate until fetal death occurs. Contractions are quite frequent (more often than 1 every 2 min). Notice that the recovery phase of the deceleration becomes increasingly more peaked with diminished variability. Immediately after the peaked accelerations, decelerations, probably late, appear. As the condition of the fetus deteriorates, the accelerative peaks become more isolated. As this pattern progresses, the variability in the decelerations between the peaks diminishes. Ultimately the baby cannot tolerate this frequency of contractions; the baseline rate falls, decelerations of indefinable character supervene, variability disappears, and the heart rate trails off to fetal death. **(E)** Illustrates the deterioration of variable decelerations in the second stage of labor with the fetus in the OP position and relentless maternal pushing with frequent uterine contractions. The amplitude and duration of the decelerations vary considerably, but the baseline remains stable and variability persists. With the onset of the second stage and maternal expulsive efforts (17M), the decelerations increase in amplitude and duration. Note that the amplitude of the deceleration results mostly from the rise in the baseline rather than a lower nadir. For the most part, the baseline variability remains intact. While the progressive rise in the baseline rate suggests a deterioration in fetal “reserve,” the maintenance of variability suggests that the fetus is both able to compensate and remain neurologically intact. In the face of this high frequency of uterine contractions and the rising fetal baseline, it seems reasonable to reduce uterine activity and periodically restrain the mother from pushing and allow the fetus ample time to recover. Scaling−30 bpm/cm—vertical, 3 cm/min—horizontal—each panel is 16 min wide.

Variable decelerations (Figures 8A–D) usually are identified as abrupt drops and returns in the heart rate, reaching a nadir within 30 s, and having no consistent relationship to the timing or amplitude of the contraction. They are often classified according to their duration, amplitude, and “typicality.” The latter refers to the pattern of return from the nadir of the deceleration to the baseline. Classically, variable decelerations have been attributed to umbilical cord compression. This is the most reasonable explanation in the first stage of labor prior to rupture of the membranes and sometimes afterward. However, with membranes ruptured and especially with early decelerations in the first stage of labor and maternal pushing in the second stage of labor, head compression is the more likely mechanism of this deceleration pattern (72).

In a deteriorating fetus with variable decelerations, abnormal recovery signals an increased potential for brain ischemia related to compromised cerebral perfusion from head compression. The increased ICP may elicit the Cushing mechanism (see Cranial Compression), which raises systemic arterial pressure, at least transiently, to restore cerebral blood flow and O_2_ delivery. With further deterioration, the decelerations become larger, baseline heart rate rises, variability decreases, and the recovery from the deceleration is often retarded. This late component becomes (Figure 8C) more obvious and the recovering heart rate may take on an abrupt, peaked appearance at the end of a variable deceleration followed by a late deceleration—an ominous commentary usually leading to fetal deaths (Figure 8D) sometimes with added features of a sinusoidal, checkmark, or sawtooth patterns.

These heart rate perturbations can best be understood by a pattern that has been called “subacute hypoxia.” (45) (Figure 8E). It consists of large, repetitive, and prolonged variable decelerations (decreasing >60 bpm, duration >90 s) with rising or unstable baseline rate, and reduced uterine rest time between contractions and late decelerations when there is sufficient time before the next contraction. Progressive fetal acidosis accompanies the pattern. Presumably, the compromised uterine blood flow from the frequent contractions (and often pushing) and the peripheral vasoconstriction are the root cause of the pH decline. Decreased uteroplacental flow and head compression often coexist with this kind of pattern. Promptly identifying and responding to this pattern is essential (cessation of pushing, reduction of contraction frequency, preparation for operative delivery). Ideally, this pattern reflecting advanced ischemia/asphyxia should not be allowed to develop.

### Prolonged Decelerations/Bradycardia

When the baseline FHR is below 110 bpm, it is considered persistent bradycardia. Certain normal fetuses (especially those that are postdate) may have basal rates <110–120 bpm and be normally oxygenated. Babies with congenital complete heart block have persistent rates in the 60 s and are also usually normally oxygenated, although about one-third have associated anomalies.

The term bradycardia has also been applied to decelerations that persist beyond 10 min. We much prefer the term prolonged deceleration in that it reflects the presence of a previously higher rate.

**Figure 9:**
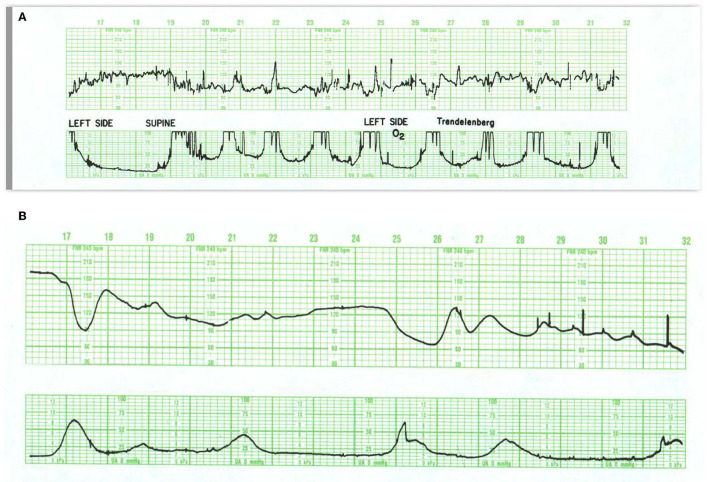
**(A)** Illustrates prolonged decelerations during second stage pushing. At 20M, the combination of frequent contractions, compulsive, expulsive efforts, and descent produce marked and dramatic change in the heart rate. Despite this, the fetus maintains abundant variability. Although a number of maneuvers were tried, including repositioning to right and left sides, Trendelenburg position, and the administration of oxygen, none influenced the heart rate pattern. The more appropriate maneuver would have been to have the patient stop pushing. The infant was delivered 30 min later and pursued a benign neonatal course. **(B)** Illustrates acute deterioration in a chronically affected fetus. Initially, this FHR pattern consists of absent variability, baseline tachycardia, variable deceleration with overshoot. From then on, the fetus can no longer maintain a stable baseline rate; the baseline falls, the deceleration at 25M evolves into a series of slow oscillations, and the fetus dies. While intervention is required, there is little evidence that intervention will change the outcome. In addition, cesarean section imposes some risk to the mother. Scaling−30 bpm/cm—vertical, 3 cm/min—horizontal—each panel is 16 min wide.

Prolonged decelerations may be caused by arrhythmias, drugs such as beta-adrenergic blockers, and rarely, prolonged hypoglycemia or hypothermia. In the previously normal fetus, a prolonged deceleration often appears in response to sudden events that require immediate intervention, such as placental abruption, uterine rupture, and umbilical cord prolapse. Prolonged decelerations can also occur with a long tetanic contraction or excessive uterine activity associated with prolonged pushing, especially with the head in the occiput-posterior position (Figures 3B, 9A). These patterns associated with pushing and excessive uterine activity are usually avoidable. Here, resolution may be anticipated with lateral positioning, reduction in uterine activity, and moderation of pushing.

The decision to “outrun fetal distress” by urging the patient with a prolonged deceleration to push exuberantly can only increase the risks of diminished oxygenation and cerebral hypoperfusion. A better tactic is to be guided by the response of the fetus to the contraction before further pushing. In the previously abnormal tracing, a prolonged deceleration often represents severe deterioration in fetal wellbeing—a step on the road to death (73) (Figure 9B). While the same provocative conditions may be present, recovery is unlikely and immediate delivery is warranted.

Recently, a number of publications have endorsed the predictive value of “deceleration area” and shown a relationship of deceleration area with both low UApH and subsequent neonatal encephalopathy irrespective of UApH (74, 75). Deceleration area is difficult to obtain visually, and as yet, there is no study of its prospective (computerized) use in a clinical setting As will be clear from the discussions in this paper, we suggest that the prevention of acidemia is more important than its prediction and that other features accompanying even prolonged decelerations (e.g., tachycardia) can assist in evaluating the true significance of this parameter (74).

It is important to recognize that confusion between the maternal and FHR may sometimes lead to the inappropriate diagnosis of fetal bradycardia. Consequently, whenever a deceleration appears to be persistent, confirmation should be obtained by auscultation or ultrasonography (see below).

## Uterine Activity

The response of CTG patterns to contractions is a fundamental feature in assessing the threat of fetal decompensation and injury (76–79). Unfortunately, identifying normal and excessive uterine activity is mired in syntactical differences and uncertain pathophysiology. Like the limits that govern automobile speeds, it is easy to declare an upper limit, but difficult to define a level of uterine activity that is invariably associated with adverse outcomes. Uterine hyperstimulation was previously defined by excessive frequency or prolonged duration of contractions or elevated baseline uterine tone (>20 mm Hg) or, more recently, diminished “rest time” the proportion of time without contractions or patient pushing (normally >50%) (80, 81). In 2008, ACOG replaced “hyperstimulation” with “tachysystole,” defined as >5 contractions per 10 min, averaged over 30 min with no reference to the other features in Table 3 (14). Waiting 30 min, however, before moderating excessive contractility seems potentially risky when even five contractions in 10 min produce a rapid reduction in fetal oxygen saturation (82). Excessive contractility does not reliably shorten labor (82–84). The presence of excessive contractility should preclude identifying the CTG tracing as normal.

## Fetal Tolerance to Hypoxia/Ischemia

Much of our knowledge of fetal tolerance to hypoxia and ischemia derives from studies in sheep and monkeys and has been summarized in recent reviews (11, 31, 85). These models have revealed that normal term fetuses are equipped with robust metabolic and cardiovascular and neurogenic defenses against the expected hypoxemic and mechanical forces of labor and enter labor with a large capacity for surviving acute oxygen deprivation or cerebral ischemia. While experimental animals are reasonable surrogates for models of acute asphyxia in the human fetus in advanced labor, they are quite poor at understanding the mechanical forces acting on the human fetal head during labor.

### Cardiovascular Adaptations—Decelerations and Their Recovery

Before dealing with the responses to hypoxia/ischemia, it is well to consider the unique importance of uterine contractions in the evaluation of fetal wellbeing. Uterine contractions, decrease uteroplacental blood flow (and fetal oxygen delivery) proportional to their intensity and duration (86, 87). By limiting oxygen availability, contractions have the potential to uncover problems of homeostasis earlier in response to contractions than between contractions. This role is played by the appearance of decelerations when critical levels of CBF and/or oxygenation are present. Contractions also stimulate the fetus and demand a response of the fetal nervous and cardiovascular systems even without significant challenges to oxygen availability. Irrespective of any impairment of oxygen availability, contractions are accompanied by a rise in fetal blood pressure and sympathetic activation, but no decelerations. Thus, the response of the fetus to the individual contraction is the mainstay of this form of surveillance, not only for understanding its behavior but the availability of resources to meet the hypoxic and ischemic challenges to its milieu. This approach has only recently been adopted in the bio-computing literature (10).

Under normal circumstances, there is sufficient oxygen and the homeostatic cardiovascular response of the fetus that permits it to endure the stress of the contraction. If the limitation in fetal PO_2_ is more than minimal, however, a compensatory, peripheral chemoreflex is triggered (31, 88) that is mediated through the release of adenosine and other vasoactive compounds that evoke vasodilatation in vital organs and neurogenic vasoconstriction in the viscera and peripheral tissues, centralizing blood flow in order to maintain oxygen delivery to priority organs (89). It is axiomatic in fetal monitoring that decelerations in association with contractions are the first sign of compensation appearing prior to any change in the baseline rate or variability. Oxygenation may also be limited by umbilical cord compression and regionally by compression of cerebral vessels or impaired cardiac output. These will be reflected in the shape, timing, and duration of the deceleration as discussed below.

Fetal hypoxia is accompanied by delayed, periodic fall in FHR with contractions (late decelerations) as part of this compensatory process—a reflection of a drop of pO_2_ below a critical threshold. The slower rate allows increased efficiency of myocardial oxygen extraction and, at least up to a point, stroke volume increases sufficiently to maintain left ventricular output. As long as these compensatory activities are equal to the task and fetal circulation is unimpeded, acid-base status, cardiac output, and cerebral perfusion remain adequate with prompt return to homeostasis (normal FHR and variability) after the contraction.

Measured between decelerations, a rise in FHR and diminution in baseline variability from the previously normal baseline rate reflects the extent of the oxygen deprivation and the more sustained rise in sympathoadrenal release of catecholamines (90, 91). Severe fetal hypoxemia dilates the arterial supply to vital organs, fetal tissues extract a greater percentage of oxygen from hemoglobin and use circulating glucose for energy. These adaptations enrich the oxygen content of the left ventricular outflow, which forestalls the fall in pH in arterial blood supplying the brain and heart (92). As the hypoxia continues, the decelerations become larger, the rate higher (rarely exceeding 170 bpm) with absent variability.

### Metabolic Adjustments

During hypoxia, the fetus conserves oxygen for vital organs by lowering oxygen consumption (93) through diminishing activity (breathing and body movements), protein synthesis, and oxidative metabolism (89, 94, 95). Furthermore, the decline in oxygen availability shifts ATP synthesis to anaerobic glucose metabolism, and utilization of glycogen stores in the heart and liver occurs (96, 97). The fall in arterial pH and the rise in the base deficit and lactate reflect the extent of anaerobic metabolism; they diminish at a pace related to the frequency of contractions and the amount of time available between contractions for fetal recovery (98–100).

## Mechanisms of Hypoxia—Ischemia

### Umbilical Cord Compression

When perfusion is compromised by pressure on the umbilical vessels, the rapidity of the response and the pattern of deterioration differ from that described above. Reduced umbilical perfusion elicits an autonomically mediated, immediate fall in heart rate (variable deceleration) and rises in fetal blood pressure that strives to maintain flow to the placenta, the brain, the heart, and adrenal glands. The ability of the fetus to adapt to the circulatory compromise caused by umbilical cord compression depends on the frequency, completeness, and duration of the occlusion. If cord compromise is sufficiently severe, hypoxemia and acidosis will develop because occlusion decreases oxygen availability (101).

In the consideration of the effects of cord compression on the fetus, two extremes must be factored in. On the one hand, a fetal neurological injury may be induced with repetitive episodes of cord compression even without significant fetal acidemia (102). At the other extreme, experiments of prolonged relentless cord occlusion were sufficient to result in severe hypercapneic hypoxemia and acidemia with bradycardia produce major reductions in cerebral blood flow and O_2_ consumption readily proceed brain injury (94). The severity of the resulting neurological injury, however, varies considerably and importantly, according to the duration of hypoperfusion, but not to the severity of acidosis or the duration of the bradycardia (64). Importantly, the features that clearly related to the severity of the injury. These included the persistent loss of variability and tachycardia in the pattern of recovery of the FHR after removal of the occlusion (64). The effects of cord compression and head compression (see below) are enhanced if the fetus is already compromised by decreased oxygen availability during contractions and by maternal pushing.

### Cranial Compression

There is considerable controversy over the role of mechanical factors in the genesis of abnormal FHR patterns and neurological injury (103, 104). From a teleological perspective, mechanical forces exerted on the fetal head during labor and delivery would seem to be as predictable an eventuality as the hypoxemic effects of uterine contractions and should generate similar robust, protective responses. In fact, there is clear evidence of central receptors sensitive to even subtle changes in cerebral blood flow or perhaps even to astrocyte (105) detection of distortion of cerebral vessel walls that act to maintain cerebral perfusion even absent systemic acidemia (104). Furthermore, there are a collection of primitive homeostatic reflexes quite active in the term fetus (dive reflex, etc.) resulting in elevations of BP that are independent of pH, but related to factors that affect the face and the trigeminal nerve (106).

Pursuing the teleological argument one step further, it should be noted that the reflexes related to both hypoxia and head compression including the Cushing response and the dive reflex, diminish over several months after birth (107). These robust reflexes were created to deal with the unique problems of labor and delivery and not again in the same way thereafter.

Abundant clinical, pathological, and experimental evidence supports the notion of intrapartum fetal head compression as a potential cause of FHR decelerations as well as ischemic and traumatic neurological injury (108). Although such (early or variable) decelerations are not generally associated with significant fetal acidemia, neither are they always innocuous. This perspective, and its related controversy, is presented more fully elsewhere (70, 108, 109).

Both between and during contractions, the intracranial pressure (ICP) is always greater than the intrauterine pressure (IUP). This serves to protect the brain from the outside pressure but increases the blood pressure required to perfuse the brain. In the defense of a contraction-related increase in ICP, each contraction evokes a rise in the fetal BP to offset the increased ICP. Within limits, especially in the first stage of labor where the head is high, and the pressure acting on the fetal skull is uniform, this response is sufficiently protective and the modest rise in pressure is sufficient to maintain cerebral perfusion. Greater compensation is required to maintain cerebral perfusion pressure during the second stage of labor when the pressure on the fetal head may be significantly greater than the intrauterine pressure (IUP) and is no longer uniform. Maternal pushing not only increases the pressure further but changes the pressure dynamics. During pushing, pressures are higher, more abrupt, and more sustained. Failure to meet these augmented pressure demands increases the potential of impaired brain perfusion and the potential for consequent neurological injury.

In theory, direct brain ischemia impairs oxygen and substrate delivery and the circulatory elimination of metabolites. It may thereby pose a greater threat to the brain than hypoxia alone. While hypoxia (without interference with fetal circulation) modulates the degree of damage when ischemia is present, ischemia may cause injury without systemic hypoxia or acidosis—or neonatal depression. Hypoxia without ischemia probably does not cause injury (110).

### Fetal Decompensation

Prolonged, severe hypoxia leads to profound acidemia (pH < 7.0), an isoelectric electrocorticogram, deteriorating cardiovascular support of blood flow to the brain, the heart, and the placenta, and the potential for brain ischemia (31). Acidosis, reduced perfusion, and depletion of energy substrates contribute to cardiac dysfunction (43) with resulting hypotension, loss of cerebral autoregulation, and diminished cerebral blood flow (111). Under these conditions, brain perfusion increasingly favors the brainstem at the expense of the cerebrum (112). Continued, severe oxygen deprivation eventually exhausts all fetal defenses, leading to central vasoconstriction, peripheral vasodilatation, hypotension, and bradycardia. The result is multiorgan failure or death, accompanied by tell-tale, agonal FHR patterns (see below) (73).

### CTG Patterns and Neurological Injury

ACOG guidelines accept that the deterioration of a Category I (normal pattern) to a Category III pattern (decelerations with absent variability or prolonged bradycardia) may represent a severely asphyxiated fetus or one already injured, depending upon the evolution of the features (14). Category III tracings are sometimes correctable (e.g., when associated with diabetic ketoacidosis), but they generally demand immediate intervention, although severe acidosis is present in only about 50% of fetuses with these tracings (15).

**Figure 10:**
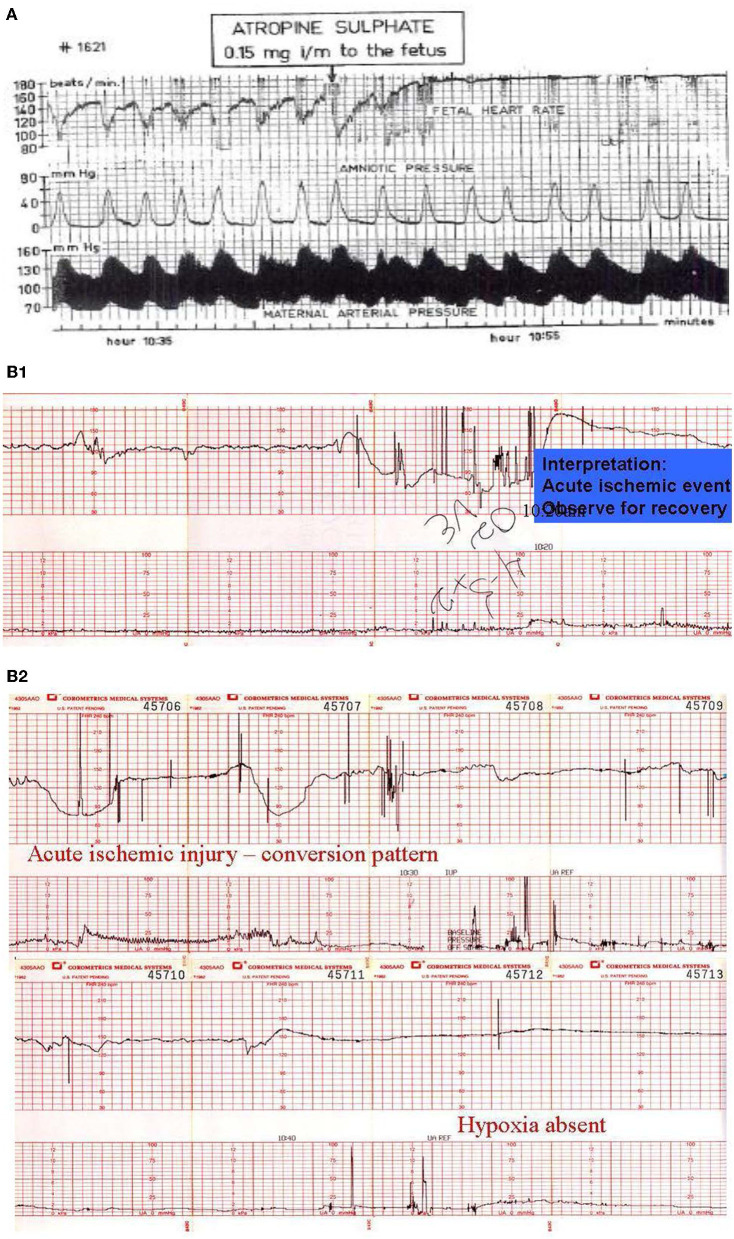
**(A)** Illustrate the effects of atropine on the fetus during the 2nd stage of labor. Note the disappearance of the variable decelerations, the rise in baseline rate and the complete loss of baseline variability following the administration of atropine directly to the fetus (note scaling factor 30 bpm/cm vertical 1 cm/min horizontal). From: Schwarcz et al. (113). Scaling−20 bpm/cm—vertical, 1 cm/min—horizontal—the panel is 60 min wide. **(B)** Illustrates the appearance of a perinatal arterial ischemic stroke and the appearance of the “conversion pattern.” Notice the initially normal tracing **(B1)** followed by the sudden appearance of a prolonged deceleration whose recovery dramatically overshoots the previous baseline heart rate of 150 bpm. The pattern evolves through several decelerations to a stable baseline with persistently absent variability and absent decelerations permitting the diagnosis of fetal neurological injury **(B2)**. Scaling−30 bpm/cm—vertical, 3 cm/min—horizontal—each panel is 16 min wide.

When the sequence of Category I or Category II to Category III occurs rapidly, accompanying changes in FHR patterns sometimes permit the confident prediction of fetal hypoxic-ischemic injury—an evolution deemed the *conversion pattern*, which probably reflects ischemic injury due to cerebral hypoperfusion to those sectors of the brain controlling vagal efferent activity. The pattern reveals a sudden rise in baseline rate, disappearance of variability, and diminution in decelerations similar to that seen with the administration of atropine (Figures 10A,B1,B2). Over time, the baseline rate may fall but moderate variability does not return (114, 115). With this pattern, the newborn usually is depressed and encephalopathic, but the umbilical pH is usually above 7.0. The severity of the accompanying neurological damage cannot be established on this basis alone, but we are unaware of a benign outcome associated with this pattern.

Whether the precursor is hypoxia or ischemia or both, the pattern of intrapartum ischemic brain injury ultimately depends on the source and severity of the ischemia. Most hypoxic and ischemic insults during labor are intermittent and contraction-related where decompensation occurs gradually, and neuronal injury occurs most frequently in the supratentorial watershed zones of the brain (the parasagittal sinus, cerebellar neocortex, and the dorsal horn of the hippocampus). The deep structures of the brain including the basal ganglia, midbrain, and medulla are usually spared. This pattern of injury is referred to on imaging as “partial, prolonged hypoxia.” It results from the sympathetically mediated redirection of brain blood flow from the carotid circulation to the vertebrobasilar arteries. By contrast, when the impairment of flow is acute and profound, as with a sentinel event (ruptured uterus, prolapsed cord, abruption), deep nuclear structures including the basal ganglia, thalamus, and midbrain, and the medulla become much more susceptible to injury because they are the most metabolically active parts of the brain. Experimental animal studies support this distinction. Even with the same total duration of hypoxia, repeated severe episodes cause greater neurologic injury than a single severe prolonged event (116). The pattern of neuronal damage is further influenced by the pre-labor metabolic and growth status of the fetus, as well as the metabolic and nutritional status of the mother (116).

## CTG Special Situations

### Abnormal Admission Tracing

Every effort should be made to identify an abnormal tracing at the start of monitoring, especially those with diminished variability and tachycardia (>150) that persists beyond 20–30 min (18). In a study of 80 cases of term HIE in Sweden, Jonsson et al. found that those patients whose initial tracing was abnormal were delivered earlier than those with normal tracings but suffered at least as high a risk of adverse outcome. This observation alone is a risk factor for adverse outcomes, tending to occur in fetuses with preexisting growth restriction, drug effect, neurological damage, or anomaly (117). Recent injury is likely if a previous non-stress test was normal (118). Asking the mother about the presence or absence of fetal movement can sometimes be helpful in estimating the time of injury (119). When this pattern is present at the outset of monitoring, it cannot be inferred that subsequent encephalopathy or permanent central nervous system injury occurred during labor. We suggest that to understand the benefits of CTG monitoring, outcome analyses must stratify patients according to the normality of the tracing on admission, sentinel events, and the urgency and timing of intervention.

### Maternal-Fetal Heart Rate Confusion

**Figure 11:**
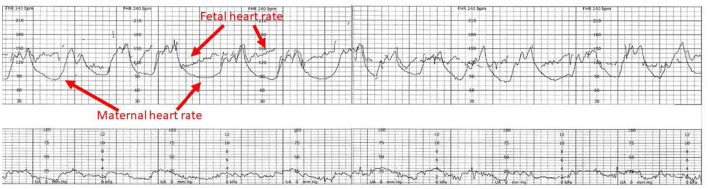
The heart rate tracing demonstrates the characteristic amplitude, height and timing of maternal accelerations associated with maternal pushing, especially when the tracing is continuous. The potential for inadvertently recording the MHR during labor has been known since the introduction of EFM. The problem is facilitated by the elevated maternal baseline, more likely in the 2nd stage of labor. The appearance of accelerations with bearing down efforts. The obstetrical care provider must recognize the potential for misinterpreting the maternal heart rate for the fetal heat rate and resolve any uncertainty. Applying the maternal transducer or have the mother cease pushing will often help resolve the issue. The application of a scalp electrode is usually definitive. Scaling−30 bpm/cm—vertical, 3 cm/min—horizontal—each panel is 16 min wide. *Tracings reprinted from Schifrin (122) (with permission from the publisher). **Tracings reprinted from Schifrin (123) (with permission from the publisher).

Occasionally, the CTG system inadvertently records the maternal heart rate (MHR) rather than that of the fetus (120, 121). The MHR may appear very similar to the FHR pattern, especially with maternal tachycardia. This is a particular risk during the expulsive phase of labor when maternal tachycardia is common, and her movement may misdirect the transducer signal (Figure 11). The Doppler-based monitor may focus on a maternal abdominal or pelvic artery to generate a heart rate tracing that may be falsely reassuring (if the mother has a tachycardia) or cause spurious concern if the MHR is normal. Of further concern is the situation in which the fetus is dead. In these unfortunate circumstances, the fetus may transmit maternal ECG voltages to a scalp electrode. The resulting MHR tracing will be mistakenly assumed to have risen from a living fetus. Similarly, ultrasound-based monitors may choose a maternal vessel and display what appears to be an FHR tracing. Newer external monitoring techniques using fetal ECG signals from the maternal abdominal wall dramatically reduce the risk of such confusion. Heightened awareness and the simultaneous recording of the MHR should be employed routinely (Figure 11).

### Indecipherable Baseline

Some FHR patterns can be difficult to interpret. In such situations, the primary objective is to determine the baseline rate and variability between contractions. These will generally emerge upon reducing contraction frequency if possible and by ceasing maternal pushing. A direct fetal scalp electrode may be required. Further assessment should take into account adjuvant methods (i.e., fetal stimulation tests), and the assessment of the frequency and severity of decelerations and their inter-deceleration patterns (i.e., normal or reduced variability, normal or increasing baseline). Delivery should be considered if resuscitative maneuvers fail to make the tracing interpretable.

## Conclusion

In this review, we have focused on current precepts and guidelines dealing with intrapartum fetal surveillance by underscoring fetal physiologic compensations to hypoxemic, ischemic, and mechanical stimuli during labor. This background provides a perspective for the interpretation of FHR patterns, for the establishment of a physiological classification, and for stratification of certain patterns in the evaluation of the role of CTG in long-term outcomes. Our approach is driven by the (preventive) notion of keeping the fetus out of harm's way by trying to restore fetal homeostasis as early as possible, not when the fetus is threatened by hypoxia or impaired cerebral blood flow. We suggest that FHR patterns provide early insight into fetal behavior, blood pressure, and cerebral blood flow, and much can be done to prevent their compromise.

The presence of normal, epochal changes in variability and accelerations and absent decelerations in the CTG excludes both hypoxic and ischemic threats to the fetus. It endorses further labor and safe delivery, but only if the uterine activity is not excessive and reasonable expectations for safe vaginal delivery exist based on labor progression. Decelerations may be tolerated as long as each recovers to a previously normal, determinable baseline rate, and variability. We emphasize that early attention to trends in FHR and in the recovery patterns of the several deceleration permits prompt remedial intervention that is critical for preventive care and good perinatal outcomes. Their likely source should be sought and corrected whenever possible.

Avoidance of excessive uterine activity, limiting maternal expulsive efforts when necessary and possible can be effective preventive actions to safeguard fetal wellbeing and reduce the need to intervene emergently. The proper analysis of CTG tracings, furthermore, permits the inference of fetal neurological injury—irrespective of pH or Apgar score—and should inform the decision to apply neonatal therapy for neuroprotection.

Furthermore, we hypothesize that we have not yet exhausted the benefits and the insights that can be gleaned from the thoughtful, informed, visual inspection of FHR and uterine contraction recordings and suggest that greater awareness and promulgation of these principles will enhance training in CTG analysis (124, 125). Current training programs reduce “head compression” to the presence of “benign,” early decelerations—with no mention of the potential consequences of the mechanical forces of labor.

Not all adverse outcomes, particularly cerebral palsy, are related to events of labor. The fetus may be neurologically compromised by events that precede labor, or that occur in the neonatal period. Furthermore, injury can occur so rapidly and unpredictably as to preclude the prevention of adverse outcomes regardless of the rapidity of intervention. However, we also recognize that preventable fetal injury does occur during labor related not only to asphyxia but also to mechanical forces of labor and delivery. We must do more to determine the long-term consequences of obstetrical care and take better advantage of the predictive information that FHR patterns provide.

## Research Recommendations

- The evaluation of CTG patterns preceding emergency intervention for fetal indications.
- The ultrasonic (Doppler) evaluation of CBF in labor both between and during contractions and with and without pushing.
- The evaluation of the role of the cessation of pushing on the recovery of concerning fetal decelerations.
- The evaluation of the role of pushing with alternate contractions (and the response of the fetus) on the incidence of abnormal CTG patterns.
- The assessment of babies with neonatal encephalopathy and pH > 7 who enter labor with normal CTG.

## Author contributions

All authors participated in the study design. BS wrote the manuscript. BK, WC, and MS critically reviewed and assisted in the revision of the manuscript. All authors contributed to the article and approved the submitted version.

## Conflict of Interest

The authors declare that the research was conducted in the absence of any commercial or financial relationships that could be construed as a potential conflict of interest.

## Publisher's Note

All claims expressed in this article are solely those of the authors and do not necessarily represent those of their affiliated organizations, or those of the publisher, the editors and the reviewers. Any product that may be evaluated in this article, or claim that may be made by its manufacturer, is not guaranteed or endorsed by the publisher.
